# Identification of tRNA-derived RNAs in adipose tissue from overweight type 2 diabetes mellitus patients and their potential biological functions

**DOI:** 10.3389/fendo.2023.1139157

**Published:** 2023-07-06

**Authors:** Jie Zhang, Yingfei Xi, Qiuping Fei, Jun Xu, Jinxing Hu

**Affiliations:** Department of Endocrinology, Hwa Mei Hospital, University of Chinese Academy of Sciences, Ningbo, Zhejiang, China

**Keywords:** tRNA-derived fragments, type 2 diabetes, overweight, fat, biomarker

## Abstract

**Background:**

Type 2 diabetes mellitus (T2DM)causes a huge public health burden worldwide, especially for those who are overweight or obese, the pain is often greater. And search for effective targets in overweight T2DM could help improve patient quality of life and prognosis. tRNA-derived RNAs (tsRNAs) are multifunctional regulators that are currently receiving much attention, but there is still a lack of knowledge about tsRNAs in overweight T2DM.

**Methods:**

T2DM patients with BMI ≥ 25 (Overweight group) and BMI< 25 (Control group) were subjected to tsRNA sequencing; differentially expressed tsRNAs in the two groups were analyzed and their expression was verified using qRT-PCR. The biological function of downstream target genes was also evaluated by enrichment analysis.

**Results:**

qRT-PCR evaluation identified a tsRNA with up-regulated expression (tRF-1-28-Glu-TTC-3-M2) and a tsRNA with down-regulated expression (tRF-1-31-His-GTG-1), both of which may be involved in metabolic and energy-related processes.

**Conclusion:**

Dysregulation of tsRNA expression in overweight patients with T2DM suggests a potential role for tsRNA in the development of T2DM.

## 1 Introduction

Type 2 diabetes mellitus (T2DM) is a cause of human suffering, with its prevalence increasing annually, affecting over 400 million people worldwide. This condition can severely compromise patients’ quality of life, lead to significant premature deaths, and place a heavy economic burden on global health resources (1, 2). Overweight or obesity, another common metabolic disorder, is now a recognized risk factor for T2DM (2). T2DM patients are primarily characterized by increased fat content around the abdomen, and this adipose tissue accelerates insulin resistance (IR) development through an inflammatory response (3). This is a key aspect of T2DM development. However, it has also been reported that lower BMI in Asian populations is associated with a higher incidence of T2DM (2), which appears to correlate with the pattern of fat accumulation. Thus, the link between T2DM and overweight is complex and warrants further exploration.

As next-generation sequencing technologies advance, an increasing number of sequencing technologies and genes are being highlighted, and tRNA-derived RNAs (tsRNAs) are gaining more attention. tsRNAs are small non-coding RNAs precisely cleaved from tRNAs, and numerous studies have shown that tsRNAs are multifunctional regulators associated with various physiological and pathological states, and can even serve as causal factors in diseases such as cancer and inherited metabolic disorders (4, 5). tsRNAs include tRNA-derived fragments (tRFs) and tRNA halves (tiRNAs), which have different lengths and biological functions (5). Some tsRNAs have been found to be involved in adipose differentiation, promoting the proliferation of preadipocytes or enhancing adipogenesis to mediate obesity (6, 7). Other studies have identified tsRNA as a key factor in the pathogenesis of diabetes and its related diseases (8), and suggested that alterations in tsRNA may be closely related to the mechanism of pancreatic β-cell death (9). Yan et al. also discovered that tsRNA may act as a new target in the treatment of diabetic foot ulcers by regulating Wnt signaling (10). We hypothesize that tsRNAs may also contribute to disease development in overweight individuals with T2DM, but the details have yet to be reported.

A BMI of ≥25 is considered overweight and obese (10). In this study, we sequenced adipose tissue from both overweight and normal-weight individuals with T2DM, identified differentially expressed tsRNAs, validated their expression, and evaluated their biological functions. Through this process, we uncovered a new perspective on the mechanisms of intervention for overweight patients with T2DM.

## 2 Methods and datas

### 2.1 Sample collection

In accordance with the Guidelines for the Prevention and Treatment of Type 2 Diabetes in China (2020 Edition), T2DM patients preparing for cholecystectomy were recruited for testing. Biochemical tests for fasting insulin, fasting glucose, fasting C-peptide, and glycated hemoglobin were completed, with the following inclusion and exclusion criteria:

①Inclusion Criteria

All patients had a primary diagnosis of T2DM, were aged 45-80 years, did not use GLP-1 agonist drugs, insulin preparations, or thiazide diuretics, were ready for cholecystectomy, had normal liver function, and had normal serum creatinine, urea nitrogen, and electrolyte levels.

②Exclusion Criteria

Excluded were patients with non-T2DM, presence of diabetic ketosis, diabetic ketoacidosis, diabetic hyperosmolar state; those using insulin, insulin analogues, dipeptidyl peptidase (DPP-IV) inhibitors, metformin, insulin sensitizers (thiazolidinediones), glucagon-like peptide (GLP-1) analogues, thyroxine, glucocorticoids, or sex hormone replacement therapy; and those with acute infections, tumors, pregnancy, or lactation.

After obtaining signed informed consent from each patient or their family, two pieces of intraoperative large omental adipose tissue, approximately 1 cm³ each, were collected for the follow-up study (registration number ChiCTR2000032718), which was performed with approval from the ethics committee of our hospital.

### 2.2 RNA extraction and pre-treatment

Patients with BMI ≥ 25kg/m^2^ were classified as the Overweight group, while those with a BMI< 25 kg/m² were designated the Control group. Three tissue samples were selected from each group, and total RNA was extracted using TRIzol. The concentration and purity of total RNA in each sample were measured with a NanoDrop instrument and sent to Aksomics Inc. for testing.

### 2.3 Sequencing and QC of tsRNA

Following the same procedure as reported in previous studies (11), interfering RNA modifications were removed, and total RNA from the samples was sequentially ligated to 3’ and 5’ small RNA adaptors. cDNA was synthesized and amplified using Illumina reverse transcription and amplification primers. PCR-amplified fragments were obtained, purified from PAGE gels, and sequenced with an Agilent 2100 instrument. Libraries were quantified, denatured, and diluted, and the diluted libraries were loaded onto the Illumina Next 500 system for sequencing.

Quality control of sequencing is performed using quality scores (Q), where $Q=-10\times \log_{10}P$ Mass fractions were plotted, and a Q > 30 was considered high quality. The reads were trimmed, converted to FASTA format, and they were also compared to matured and precursor tRNA sequences from the GtRNAdb (12, 13) database and tRNAscan-SE (14) software. After excluding any predicted intronic sequences, a 3’-terminal ‘CCA’ was appended to each tRNA. Next, 40 nucleotides of flanking genomic sequence were included on either side of the original tRNA sequence (15). With matching reads using Bowtie software (16), the remaining reads were finally aligned with miRDeep2 (17). The expression levels of the tsRNA were assessed based on the mapping reads and finally expressed using CPM (Counts Per Million), $CPM=\frac{10^{6}\times Count}{N}$

### 2.4 qRT-PCR

Among the differentially expressed tsRNA, those with higher CPM were selected for detection. cDNA was synthesized by tRF&tiRNA Kit (Arraystar) and subjected to qRT-PCR using 2X PCR master mix in the QuantStudio™ system (Applied Biosystems). See **Table 1** for primer details.

**Table 1:** Primer sequences and other details.

Gene	Sequences (5’-3’)		Temperature (°C)	Length (bp)
U6	F	GCTTCGGCAGCACATATACTAAAAT	60	89
	R	CGCTTCACGAATTTGCGTGTCAT		
tRF-1-28-Glu-TTC-3-M2	F	AGTCCGACGATCTCCCTGGT	60	48
	R	TCCGATCTCGAATCCTAGCC		
tRF-54-75-Ala-CGC-1-M4	F	AGTCCGACGATCTCGATCCC	60	46
	R	CTCTTCCGATCTTGGTGGAGA		
tRF-1-31-His-GTG-1	F	GATCGCCGTGATCGTATAGTG	60	47
	R	CTCTTCCGATCTCGCAGAGTA		

### 2.5 Prediction of target genes and their potential functions

The GtRNAdb database was used to identify the position of tsRNAs in the corresponding tRNAs. Target genes were predicted using TargetScan and miRanda software, and the R package clusterProfiler was employed to determine the potential function of these target genes.

### 2.6 Statistics

Expression levels between samples were analyzed using principal component analysis (PCA) was employed to explore category differences between samples. Differentially expressed tsRNAs at nominal significance were analyzed using the R package edgeR (18) with the following screening conditions: abs(FC)>1.5 and P< 0.05. Hierarchical clustering of differentially expressed tsRNAs was performed using the R package heatmap2. PCA was performed to explore sample categories based on tsRNA CPM (samples without replicates were not available), and plots were generated using the R package scatterplot3d.

## 3 Results

### 3.1 tsRNA distribution in the adipose tissue

**Figure 1:**
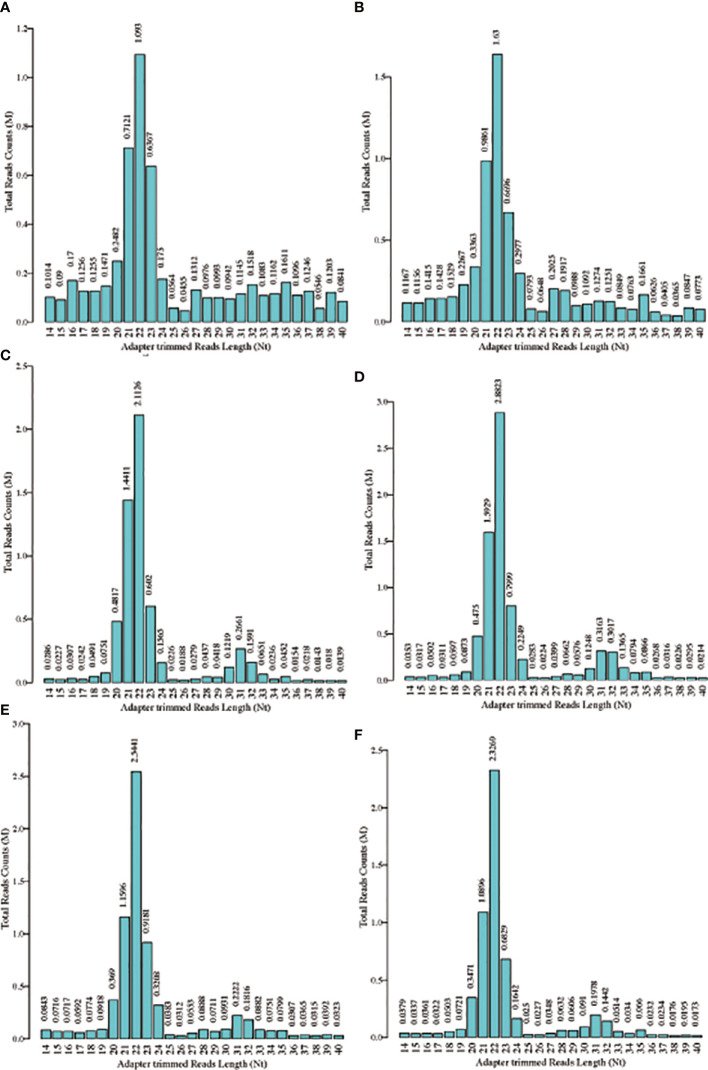
Length distribution for each sample. **(A-C)** Read lengths of the three Overweight group samples; **(D–F)** Read lengths of the three Control group samples.

**Figure 2:**
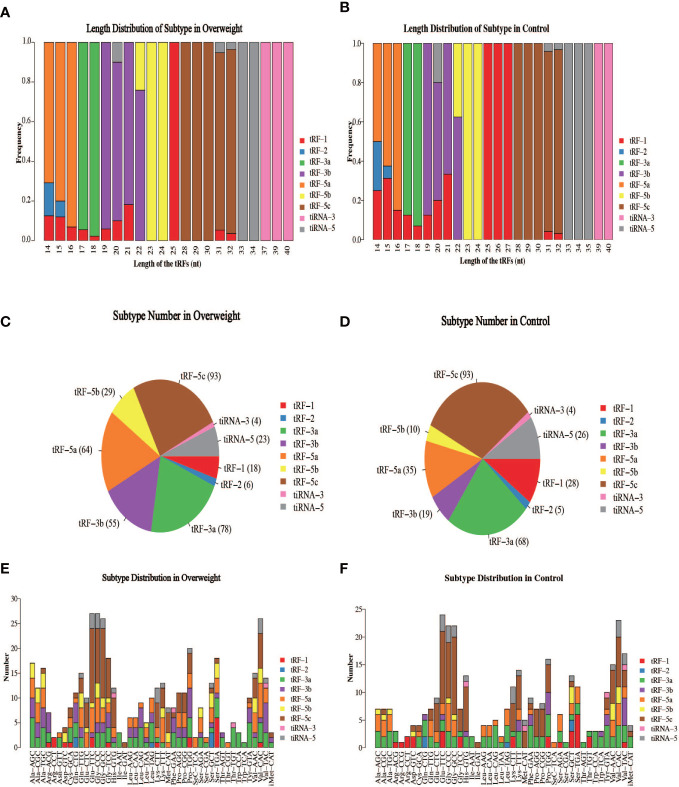
Distribution of individual isoforms of tsRNA in the two groups of samples. **(A, B)** Frequency of each isoform between Overweight and Control groups; **(C, D)** Number of each isoform in Overweight and Control groups; **(E, F)** Number of each isoform between Overweight and Control groups.

The schematic diagram of the design and main findings of this study is shown in **Supplementary Figure 1**. The general clinical characteristics of the enrolled patients are detailed in **Table 2**. The proportion of high-quality bases in each sample was >90%, and the read lengths for each sample are shown in **Figure 1**. tsRNA is known to have multiple isoforms: tRF-1, tRF-3b, tiRNA-5, tRF-5a, tRF-3a, tRF-5b, tiRNA-3, tRF-5c, and tRF-2. The frequency and number of each isoform were calculated for both the Overweight and Control groups based on the corresponding tRNA site and sequence length (**Figures 2A–D**), and the number of different tsRNA isodecoders differed between the two groups (**Figures 2E, F**).

**Table 2:** Patients’ general clinical characteristics (Mean ± SD) [n(%)].

Sample	Groups	BMI (,25kg/m^2^)	Age (, Year)	Gender (, Female)	SBP (, mmHg)	DBP (, mmHg)	Cr (, umol/L)	eGFR (, mL/min)	ACR (, mg/mmol)	FBG (, mmol/L)	HbA1c (, mmol/L)	TC (, mmol/L)	TG (, mmol/L)	LDL (, mmol/L)	HDL (, mmol/L)
1	Overweight	25.68	57	Male	127	84	74.2	100.4	9.4	10.89	10.1	4.09	2.17	2.41	0.93
2	Overweight	27.94	72	Female	121	63	40.5	85.9	139	6.56	9	3.8	1.54	2.3	0.92
3	Overweight	26.4	66	Male	146	76	53.7		5.5		6.3	4.67	1.11	2.55	1.64
4	Overweight	26.02	62	Male	113	73	73.1	102.5		14.5					
5	Overweight	28.9	69	Female	160	93	58.9			5.71	7	6.13	2.75	3.68	1.45
6	Overweight	26.76	71	Male	118	86	189.4	30.2	15.2	13.7	11.2	4.01	2.84	2.5	0.8
7	Control	20.81	48	Female	102	76	53				7.4	2.23	0.87	1.25	0.66
8	Control	17.6	66	Male	98	58	49.4	108.4		12.03					
9	Control	24.14	78	Female	150	65	63.1	82.5	22	5.28	8.1	5.31	1.67	2.74	1.31
Mean ± SD	All (n=9)	25.14 ± 3.74	65.44 ± 8.89	4 (44.44%)	126.11 ± 21.65	74.89 ± 11.54	72.81 ± 45.05	84.98 ± 28.64	38.22 ± 56.68	9.81 ± 3.90	8.44 ± 1.76	4.32 ± 1.24	1.85 ± 0.77	2.49 ± 0.71	1.10 ± 0.37
	Overweight (n=6)	26.95 ± 1.23	66.17 ± 5.78	2(33.33%)	130.83 ± 18.30	79.17 ± 10.68	81.63 ± 54.28	79.75 ± 33.85	42.28 ± 64.61	10.27 ± 4.02	8.72 ± 2.06	4.54 ± 0.43	2.08 ± 0.75	2.69 ± 0.56	1.15 ± 0.37
	Control (n=3)	20.85 ± 3.27	64.00 ± 15.10	2(66.67%)	116.67 ± 28.94	66.33 ± 9.07	55.17 ± 7.10	/	/	/	/	/	/	/	/

BMI, body mass index; SBP, systolic blood pressure; DBP, diastolic blood pressure; Cr, creatinine; eGFR, glomerular filtration rate; ACR, urine albumin-to-creatinine ratio; FBG, fasting blood glucose; HbA1c, glycated hemoglobin; TC, Total cholesterol; TG, triglyceride; LDL, low-density lipoprotein; HDL, High-density lipoprotein.

### 3.2 Presence of differentially expressed tsRNA in the overweight group compared to the control group

**Figure 3:**
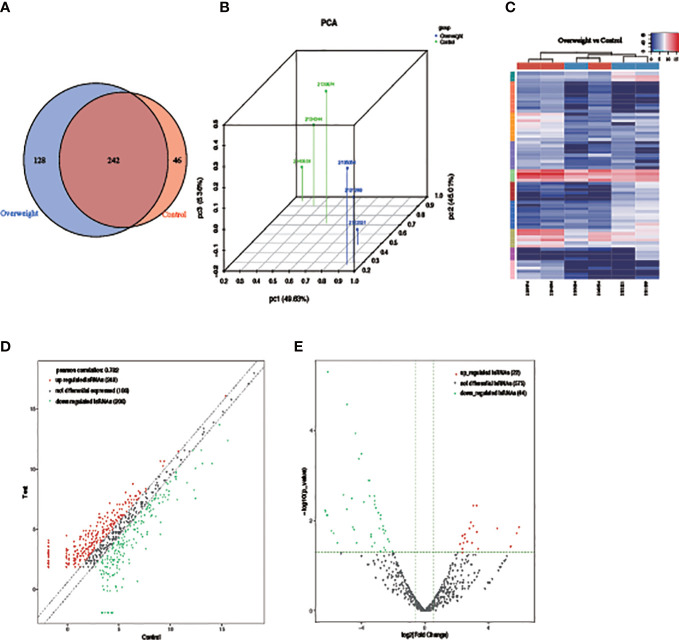
Differences in tsRNA expression between two groups of samples. **(A)** PCA used to explore sample categories; **(B)** Venn diagram showing the number of expression profiles; **(C)** heat map showing clustering of differentially expressed tsRNAs; **(D)** scatter and **(E)** volcano plot showing the results of differential analysis of tsRNAs.

PCA results showed notable differences in the expression profiles of the two groups of samples based on the clustering of tsRNA expression profiles (**Figures 3A, B**). There were 242 tsRNAs commonly expressed in both sample groups, and 128 were specifically expressed in the Overweight group compared to the Control group. Samples below the expression mean are shown in blue, while red is used to represent samples above the expression mean (**Figure 3C**). A total of 22 up-regulated and 44 down-regulated tsRNAs were identified, with inter-group differences in differentially expressed tsRNAs (**Figures 3D, E**).

### 3.3 Differentially expressed tsRNAs and their corresponding target gene networks

**Figure 4:**
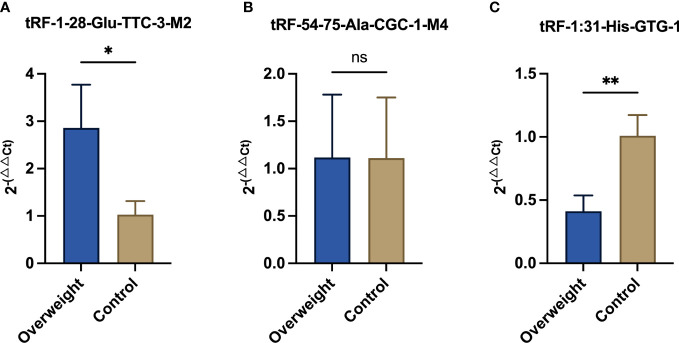
Results of qRT-PCR assays for three significantly differentially expressed tsRNAs. qRT-PCR was adopted for detecting the expression of **(A)** tRF-1-28-Glu-TTC-3-M2; **(B)** tRF-54-75-Ala-CGC-1-M4; and **(C)** tRF-1-31-His-GTG-1 between the two groups of samples. * means P<0.05; ** means P<0.01; ns means not statistically significant.

Among the 66 differentially expressed tsRNAs, those with higher expression abundance and length of 18 bp or more in each sample were selected for qRT-PCR, as detailed in **Table 3**. As shown in **Figure 4**, compared to diabetic patients with normal BMI, overweight diabetic patients exhibited upregulation of tRF-1-28-Glu-TTC-3-M2 expression and downregulation of tRF-1-31-His-GTG-1 expression in adipose tissue (both P< 0.05). Further details of these two tsRNAs are given in **Supplementary Table 1**.

**Table 3:** Data details of the significantly differentially expressed tsRNA.

tsRNA	tRF-54-75-Ala-CGC-1-M4	tRF-1-28-Glu-TTC-3-M2	tRF-1-31-His-GTG-1
Sequence	TCGATCCCCGGCATCTCCACCA	TCCCTGGTGGTCTAGTGGCTAGGATTCG	GCCGTGATCGTATAGTGGTTAGTACTCTGCG
Type	tRF-3b	tRF-5c	tRF-5c
Length	22	28	31
log2FC	3.082	3.246	-3.144
Test CPM	7.218	6.351	12.425
Control CPM	4.135	3.104	15.569
p	0.004	0.005	0.004

### 3.4 Prediction and potential function of target genes

**Figure 5:**
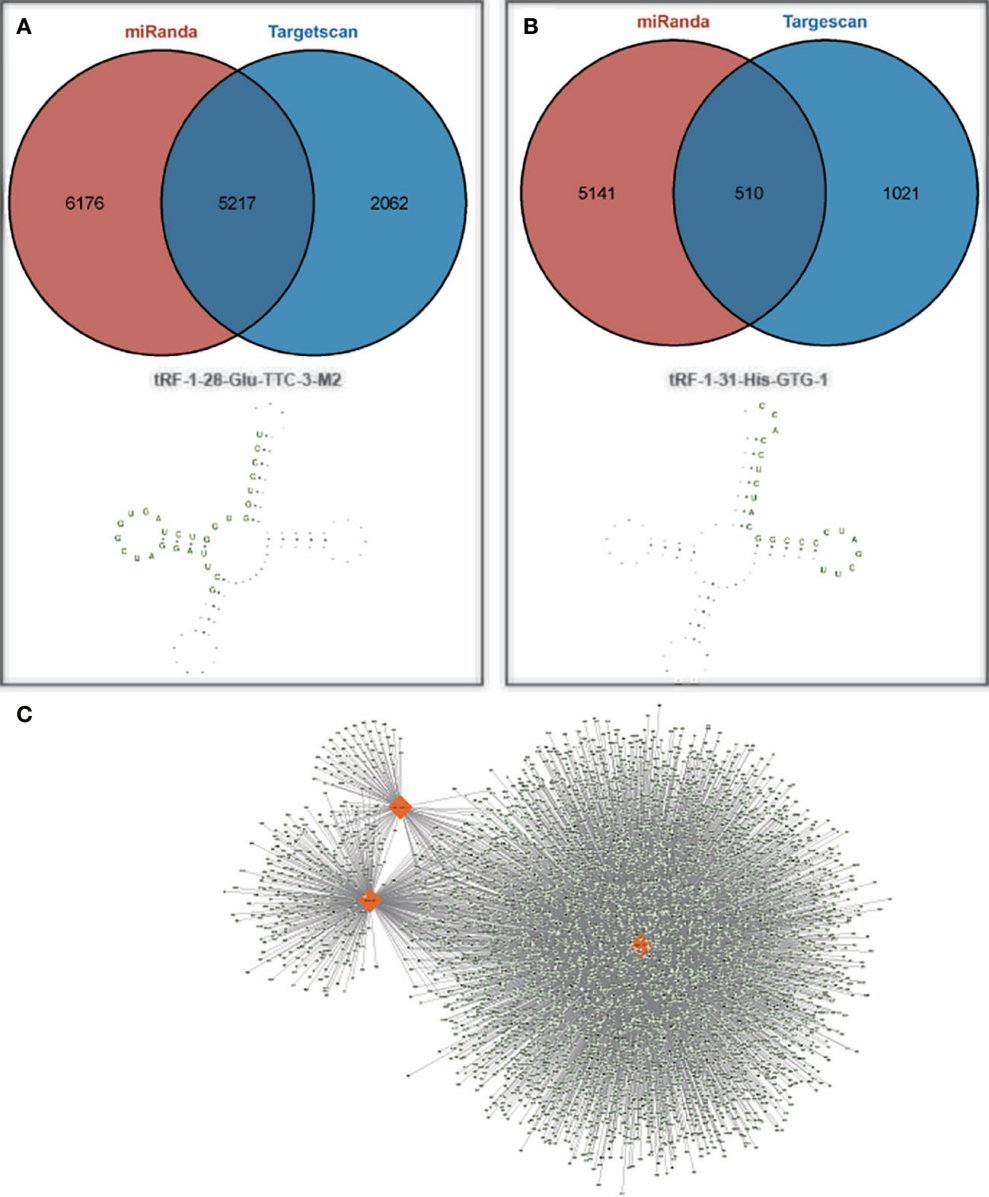
Target genes of tRF-1-28-Glu-TTC-3-M2 as well as tRF-1-31-His-GTG-1. **(A, B)** Number of target genes and their secondary structures for tRF-1-28-Glu-TTC-3-M2 as well as tRF-1-31-His-GTG-1; **(C)** Two validated tsRNAs and their predicted target gene networks.

**Figure 6:**
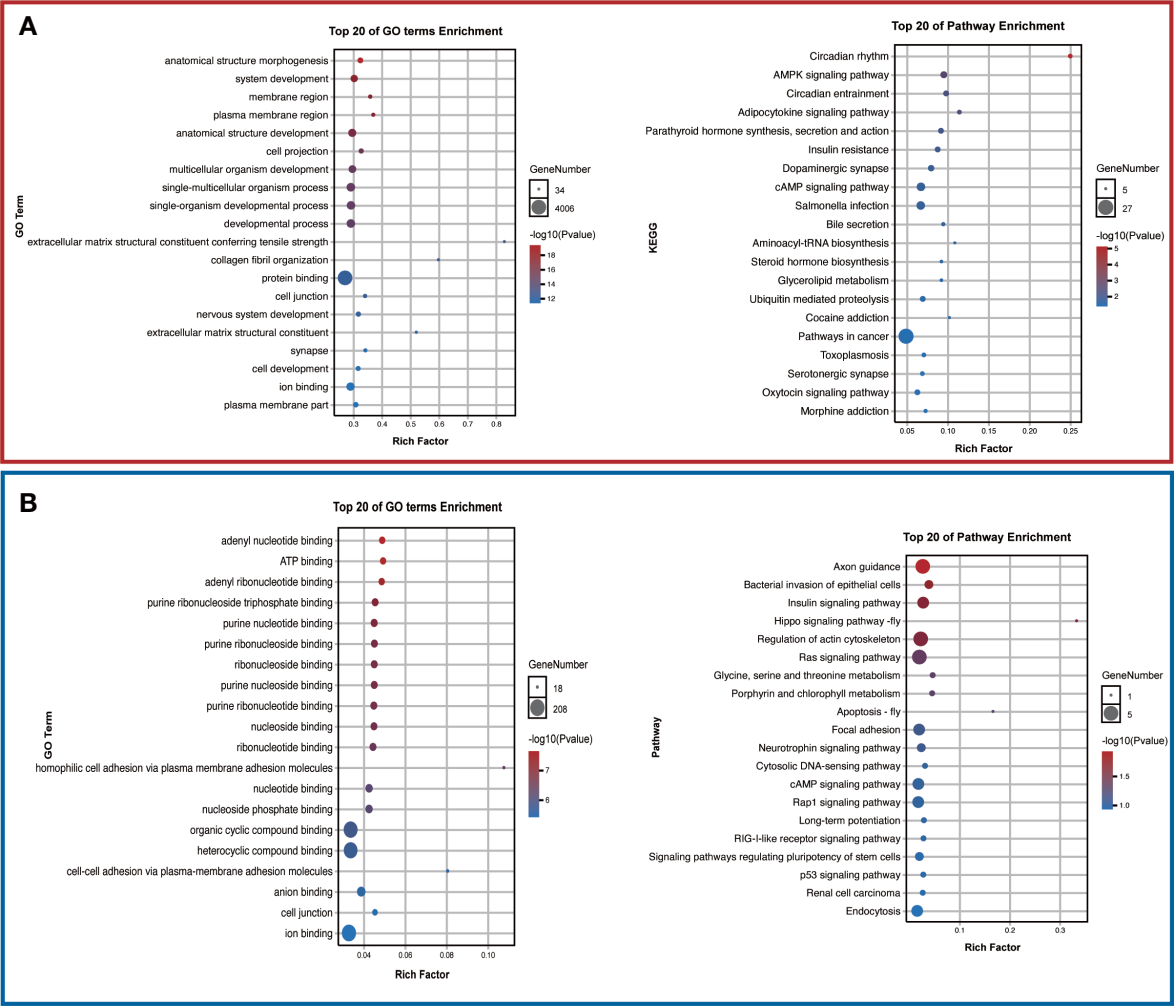
Potential functions of the validated tsRNAs. **(A)** Top 20 GO terms and pathways in tRF-1-28-Glu-TTC-3-M2 and **(B)** tRF-1-31-His-GTG-1. .

To further investigate the potential functions of these two target tsRNAs, their target genes were predicted by miRanda and targetscan databases. The predicted target genes will be intersected to obtain a total of 5725 and 510 target genes, respectively. The structures of these two tsRNAs in the corresponding tRNAs were predicted, and tRF-1-28-Glu-TTC-3-M2 and tRF-1-31-His-GTG-1 corresponding target gene networks were mapped using Cytoscape (**Figure 5**). Among these, the up-regulated tRF-1-28-Glu-TTC-3-M2 was primarily involved in biological processes such as anatomical structure morphogenesis and development and was significantly enriched in circadian rhythm and AMPK signaling (**Figure 6A**). In contrast, as shown in **Figure 6B**, the down-regulated tRF-1-31-His-GTG-1 was primarily enriched in signaling pathways such as axon guidance, insulin, and was implicated in biological processes such as adenylate binding and ATP binding.

## 4 Discussion

Obesity or being overweight has consistently been identified as the strongest trigger for T2DM, yet there are still some individuals with T2DM who have normal BMIs (19). Although islet beta-cell disorder may be due to obesity, it is actually caused by insulin resistance (IR) itself (20). Overweight, beginning in childhood and persisting into adulthood, has been suggested as a risk factor for T2DM in adults (21). In contrast, relatively lean individuals with reduced insulin sensitivity or IR can lead to fat accumulation, and T2DM may be considered the ‘cause’ of obesity (22). Nonetheless, a strong association between being overweight or obese and T2DM persists, with statistics from a UK study showing that over 80% of people with T2DM have a BMI ≥25 (23),and that in overweight individuals, a sustained increase in BMI leads to increased mortality from T2DM (24). Regrettably, there has been no progress in controlling overweight prevalence in the population over the last decade or so, even as the number of overweight individuals continues to rise and being overweight becomes nearly the population norm (8). In patients with T2DM with a BMI ≥15, appropriate weight loss significantly improves abnormally elevated blood glucose, lipids and blood pressure, enabling patients to minimize exposure to cardiovascular disease risk factors (25). Additionally, a recent study found that weight loss minimizes the occurrence of diabetic complications, and thus, appropriate weight loss or maintenance of a moderate weight, along with glucose-lowering therapy in patients with T2DM, can significantly address the underlying diseas (26).

Small nuclear RNAs (snRNAs) play a role in regulating biological developmental processes and disease pathogenesis as regulators of gene expression (27). tRNA degradation or cleavage produces large amounts of small non-coding RNAs, known as tsRNAs (4, 27). tsRNAs are involved in regulating protein homeostasis, are associated with various aspects of cellular metabolism and modes of programmed cell death, and control the expression of gene post-transcriptional genes (4). Recent sequencing data have demonstrated that tsRNA is widely expressed in the central nervous system and is increased abnormally when neuronal and protein homeostasis is imbalanced, serving as a novel biological marker for the diagnosis and treatment of neurological dysfunction (28). Moreover, the function of tsRNAs in human cancer is beginning to be increasingly recognized. tsRNAs may mediate the biological processes of cancer cells through their involvement in post-transcriptional regulation, and are new targets for cancer drug therapy, diagnosis and prognosis (29, 30).

tsRNAs are involved in adipocyte fate and function (5), and a growing number of studies suggest that tsRNAs are involved in different metabolic diseases and may play a key role in the development of metabolic diseases including T2DM (31). In the absence of interference from extrinsic factors, tsRNAs may independently influence the intergenerational inheritance of metabolic phenotypes in fertilized eggs in the form of miRNAs (32). tsRNAs may mediate metabolic disorders by participating in adipogenesis, degeneration, etc. Cristina et al. (9) showed that a TRMT10A deficiency leads to tRNA fragmentation, which induces pancreatic β-cell death, leading to the development of diabetes mellitus. However, there are still few studies related to tsRNA in T2DM. Our current study focused on overweight T2DM patients with high and low BMI and the differential expression profiles of tsRNA between their adipose tissues, in order to fill in the gaps in the knowledge of tsRNA in the context of overweight T2DM.

Our results showed that among 66 differentially expressed tsRNAs, as identified by qRT-PCR, tRF-1-28-Glu-TTC-3-M2 as well as tRF-1-31-His-GTG-1 were upregulated and downregulated in overweight T2DM patients, respectively. These two tsRNAs belong to the same tRF-5c family. A study by Gu et al. in which tsRNAs were sequenced in subcutaneous fat from fat and lean pigs found that tsRNAs were not produced by random cleavage of tRNAs, that tRF-5c was the predominant type of tsRNA in differential lipid deposition, and that tRF-5c may be inextricably linked to lipid metabolism (33). The tRF-1-28-Glu-TTC-3-M2 was found to be associated with circadian rhythm and AMPK signaling by KEGG enrichment analysis. It is well known that circadian rhythm disorders are the main cause of metabolic dysfunction in the body and that once the temporal signal is dysregulated, the endocrine system adapts to secrete hormones such as cortisol, melatonin and glucagon, affecting IR and systemic metabolism to the extent that metabolic diseases such as obesity and diabetes develop (34, 35). AMPK is a highly nutrient-responsive energy sensor whose activity is directly impacted by obesity or overweight, resulting in adverse changes in the body’s metabolism, especially in obese type 2 diabetes patients (36). Furthermore, AMPK takes a crucial part in regulating adipose tissue metabolism and development, and targeting AMPK is an effective strategy for lipid metabolism and energy metabolism in patients with T2DM (37). We conjecture that tRF-1-28-Glu-TTC-3-M2 may be involved in disease progression in overweight T2DM patients by regulating circadian rhythms or (and) signaling such as AMPK, providing a reference for subsequent precise treatment of T2DM. As for tRF-1-31-His-GTG-1, a key tsRNA with down-regulated expression, the current study found that it was significantly differentially expressed between the two groups of samples and highly correlated with signals, such as insulin and biological processes such as adenylate binding and ATP binding. This also supports the existence of differences in energy metabolism and insulin function between T2DM patients with different BMIs, but further experiments are needed to validate this.

As this is a preliminary exploration of tsRNA expression in T2DM, there are limitations to the study. Firstly, more samples need to be included that complement the expression of tsRNA in T2DM patients at all BMI stages. Secondly, the specific role of tsRNAs in overweight T2DM can be refined by targeting important biological functions in tsRNAs and finding key target genes. Finally, the specific mechanisms of key tsRNA regulation in T2DM patients are still not well understood and more *in vivo* and ex vivo experiments are needed to validate the biological functions of tsRNAs on adipocytes.

In summary, we analyzed tsRNA expression in overweight and normal weight T2DM patients, screened two hub tsRNAs: tRF-1-28-Glu-TTC-3-M2 as well as tRF-1-31-His-GTG-1, and evaluated their potential functions in T2DM, providing a theoretical basis for the role of tsRNAs in T2DM.

## Data availability statement

This study analysed publicly available datasets. The complete data for this study is stored in the Dryad Digital Repository, this data can be found here: https://datadryad.org/stash/share/T6rUqNJanSB3HENPPIFo5WC4ypbxZMXuDY7D597e6Ec.

## Ethics statement

The studies involving human participants were reviewed and approved by ChiCTR2000032718. The patients/participants provided their written informed consent to participate in this study.

## Author contributions

All authors listed have made a substantial, direct, and intellectual contribution to the work, and approved it for publication.

## Supplementary material

Supplementary Figure 1 Flowchart T2DM patients and control subjects underwent tsRNAs sequencing. Differential expression of tsRNAs was analyzed, and twotsRNAs (tRF-1-28-Glu-TTC-3-M2 and tRF-1-31-His-GTG-1) were identifiedwith potential roles in metabolic and energy-related processes. This highlights the possible influence of tsRNA dysregulation in the pathogenesis of T2DM.

## References

[1] Khan MAB Hashim MJ King JK Govender RD Mustafa H Al Kaabi J. Epidemiology of type 2 diabetes - global burden of disease and forecasted trends. J Epidemiol Glob Health (2020) 10(1):107–11. 10.2991/jegh.k.191028.001 PMC7310804 32175717

[2] Tinajero MG Malik VS. An update on the epidemiology of type 2 diabetes: a global perspective. Endocrinol Metab Clin North Am (2021) 50(3):337–55. doi: 10.1016/j.ecl.2021.05.013 34399949

[3] Galicia-Garcia U Benito-Vicente A Jebari S Larrea-Sebal A Siddiqi H Uribe KB. Pathophysiology of type 2 diabetes mellitus. Int J Mol Sci (2020) 21(17). doi: 10.3390/ijms21176275 PMC7503727 32872570

[4] Li S Xu Z Sheng J. tRNA-derived small RNA: a novel regulatory small non-coding RNA. Genes (Basel) (2018) 9(5). doi: 10.3390/genes9050246 PMC5977186 29748504

[5] Pan Q Han T Li G. Novel insights into the roles of tRNA-derived small RNAs. RNA Biol (2021) 18(12):2157–67. doi: 10.1080/15476286.2021.1922009 PMC8632077 33998370

[6] Wang T Cao L He S Long K Wang X Yu H. Small RNA sequencing reveals a novel tsRNA-06018 playing an important role during adipogenic differentiation of hMSCs. J Cell Mol Med (2020) 24(21):12736–49. doi: 10.1111/jcmm.15858 PMC7686998 32939933

[7] Shen L Tan Z Gan M Li Q Chen L Niu L. tRNA-derived small non-coding RNAs as novel epigenetic molecules regulating adipogenesis. Biomolecules (2019) 9(7). doi: 10.3390/biom9070274 PMC6681357 31336727

[8] Zhang X-T Mao Z-Y Jin X-Y Wang Y-G Dong Y-Q Zhang C. Identification of a tsRNA contributor to impaired diabetic wound healing via high glucose-induced endothelial dysfunction. Diabetes Metab Syndrome Obes (2023) 16:285–98. doi: 10.2147/DMSO.S379473 PMC9899021 36760596

[9] Cosentino C Toivonen S Diaz Villamil E Atta M Ravanat JL Demine S. Pancreatic β-cell tRNA hypomethylation and fragmentation link TRMT10A deficiency with diabetes. Nucleic Acids Res (2018) 46(19):10302–18. doi: 10.1093/nar/gky839 PMC6212784 30247717

[10] Caballero B. Humans against obesity: who will win? Adv Nutr (2019) 10(suppl_1):S4–s9. doi: 10.1093/advances/nmy055 30721956 PMC6363526

[11] Wang L Liu Y Yan W Huang C Ding Z Yang J. Clinical significance of high expression of tRF-Glu-TTC-2 in prostate carcinoma and its effect on growth. Am J Mens Health (2022) 16(6):15579883221135970. doi: 10.1177/15579883221135970 36377736 PMC9673532

[12] Chan PP Lowe TM. GtRNAdb: a database of transfer RNA genes detected in genomic sequence. Nucleic Acids Res (2009) 37(Database issue):D93–7. doi: 10.1093/nar/gkn787 PMC2686519 18984615

[13] Chan PP Lowe TM. GtRNAdb 2.0: an expanded database of transfer RNA genes identified in complete and draft genomes. Nucleic Acids Res (2016) 44(D1):D184–9. 10.1093/nar/gkv1309 PMC4702915 26673694

[14] Lowe TM Chan PP. tRNAscan-SE on-line: integrating search and context for analysis of transfer RNA genes. Nucleic Acids Res (2016) 44(W1):W54–7. doi: 10.1093/nar/gkw413 PMC4987944 27174935

[15] Selitsky SR Sethupathy P. tDRmapper: challenges and solutions to mapping, naming, and quantifying tRNA-derived RNAs from human small RNA-sequencing data. BMC Bioinf (2015) 16:354. doi: 10.1186/s12859-015-0800-0 PMC4632369 26530785

[16] Langmead B. Aligning short sequencing reads with bowtie. Curr Protoc Bioinf (2010) 32(1):11.7.1–11.7.14. doi: 10.1002/0471250953.bi1107s32 PMC3010897 21154709

[17] Friedländer MR Mackowiak SD Li N Chen W Rajewsky N. miRDeep2 accurately identifies known and hundreds of novel microRNA genes in seven animal clades. Nucleic Acids Res (2012) 40(1):37–52. doi: 10.1093/nar/gkr688 21911355 PMC3245920

[18] Robinson MD McCarthy DJ Smyth GK. edgeR: a bioconductor package for differential expression analysis of digital gene expression data. bioinformatics (2010) 26(1):139–40. doi: 10.1093/bioinformatics/btp616 PMC2796818 19910308

[19] Liu W Zhou X Li Y Zhang S Cai X Zhang R. Serum leptin, resistin, and adiponectin levels in obese and non-obese patients with newly diagnosed type 2 diabetes mellitus: a population-based study. Med (Baltimore) (2020) 99(6):e19052. doi: 10.1097/MD.0000000000019052 PMC7015632 32028423

[20] Gupta D Krueger CB Lastra G. Over-nutrition, obesity and insulin resistance in the development of β-cell dysfunction. Curr Diabetes Rev (2012) 8(2):76–83. doi: 10.2174/157339912799424564 22229253

[21] Bjerregaard LG Jensen BW Ängquist L Osler M Sørensen TIA Baker JL. Change in overweight from childhood to early adulthood and risk of type 2 diabetes. N Engl J Med (2018) 378(14):1302–12. doi: 10.1056/NEJMoa1713231 29617589

[22] Malone JI Hansen BC. Does obesity cause type 2 diabetes mellitus (T2DM)? or is it the opposite? Pediatr Diabetes (2019) 20(1):5–9. doi: 10.1111/pedi.12787 30311716

[23] Daousi C Casson IF Gill GV MacFarlane IA Wilding JP Pinkney JH. Prevalence of obesity in type 2 diabetes in secondary care: association with cardiovascular risk factors. Postgrad Med J (2006) 82(966):280–4. doi: 10.1136/pmj.2005.039032 PMC2579635 16597817

[24] Whitlock G Lewington S Sherliker P Clarke R Emberson J Halsey J. Body-mass index and cause-specific mortality in 900 000 adults: collaborative analyses of 57 prospective studies. Lancet (2009) 373(9669):1083–96. 10.1016/S0140-6736(09)60318-4 PMC2662372 19299006

[25] Wing RR Lang W Wadden TA Safford M Knowler WC Bertoni AG. Benefits of modest weight loss in improving cardiovascular risk factors in overweight and obese individuals with type 2 diabetes. Diabetes Care (2011) 34(7):1481–6. doi: 10.2337/dc10-2415 PMC3120182 21593294

[26] Apovian CM Okemah J O'Neil PM. Body weight considerations in the management of type 2 diabetes. Adv Ther (2019) 36(1):44–58. doi: 10.1007/s12325-018-0824-8 30465123 PMC6318231

[27] Hombach S Kretz M. Non-coding RNAs: classification, biology and functioning. Adv Exp Med Biol (2016) 937:3–17. doi: 10.1007/978-3-319-42059-2_1 27573892

[28] Fagan SG Helm M Prehn JHM. tRNA-derived fragments: a new class of non-coding RNA with key roles in nervous system function and dysfunction. Prog Neurobiol (2021) 205:102118. doi: 10.1016/j.pneurobio.2021.102118 34245849

[29] Li X Liu X Zhao D Cui W Wu Y Zhang C. tRNA-derived small RNAs: novel regulators of cancer hallmarks and targets of clinical application. Cell Death Discovery (2021) 7(1):249. doi: 10.1038/s41420-021-00647-1 34537813 PMC8449783

[30] Xiao L Wang J Ju S Cui M Jing R. Disorders and roles of tsRNA, snoRNA, snRNA and piRNA in cancer. J Med Genet (2022) 59(7):623–31. doi: 10.1136/jmedgenet-2021-108327 35145038

[31] Liu B Cao J Wang X Guo C Liu Y Wang T. Deciphering the tRNA-derived small RNAs: origin, development, and future. Cell Death Dis (2021) 13(1):24. doi: 10.1038/s41419-021-04472-3 34934044 PMC8692627

[32] Sarker G Sun W Rosenkranz D Pelczar P Opitz L Efthymiou V. Maternal overnutrition programs hedonic and metabolic phenotypes across generations through sperm tsRNAs. Proc Natl Acad Sci U.S.A. (2019) 116(21):10547–56. doi: 10.1073/pnas.1820810116 PMC6534971 31061112

[33] Gu H Gan M Wang L Yang Y Wang J Chen L. Differential expression analysis of tRNA-derived small RNAs from subcutaneous adipose tissue of obese and lean pigs. Animals (2022) 12(24):3561. doi: 10.3390/ani12243561 36552481 PMC9774726

[34] Parameswaran G Ray DW. Sleep, circadian rhythms, and type 2 diabetes mellitus. Clin Endocrinol (Oxf) (2022) 96(1):12–20. doi: 10.1111/cen.14607 34637144 PMC8939263

[35] Pillon NJ Loos RJF Marshall SM Zierath JR. Metabolic consequences of obesity and type 2 diabetes: balancing genes and environment for personalized care. Cell (2021) 184(6):1530–44. doi: 10.1016/j.cell.2021.02.012 PMC9191863 33675692

[36] Lyons CL Roche HM. Nutritional modulation of AMPK-impact upon metabolic-inflammation. Int J Mol Sci (2018) 19(10). doi: 10.3390/ijms19103092 PMC6213547 30304866

[37] Desjardins EM Steinberg GR. Emerging role of AMPK in brown and beige adipose tissue (BAT): implications for obesity, insulin resistance, and type 2 diabetes. Curr Diabetes Rep (2018) 18(10):80. doi: 10.1007/s11892-018-1049-6 30120579

