# Supplementary figures and images for: Identification of tRNA-derived RNAs in adipose tissue from overweight type 2 diabetes mellitus patients and their potential biological functions
Source: Front Endocrinol (Lausanne). 2023 Jul 6;14:1139157. doi: 10.3389/fendo.2023.1139157 (PMC10358832; doi:10.3389/fendo.2023.1139157)

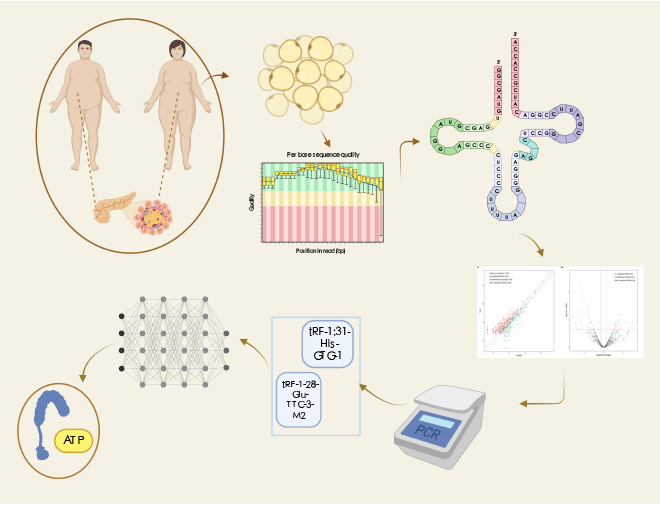

Supplement: Supplementary Figure 1 — Flowchart T2DM patients and control subjects underwent tsRNAs sequencing. Differential expression of tsRNAs was analyzed, and twotsRNAs (tRF-1-28-Glu-TTC-3-M2 and tRF-1-31-His-GTG-1) were identifiedwith potential roles in metabolic and energy-related processes. This highlights the possible influence of tsRNA dysregulation in the pathogenesis of T2DM. [file Image_1.tif]
